# Periodontitis, A True Infection

**DOI:** 10.4103/0974-777X.56251

**Published:** 2009

**Authors:** Rajiv Saini, P P Marawar, Sujata Shete, Santosh Saini

**Affiliations:** *Department of Periodontology, Rural Dental College, Loni, Maharashtra, India*; 1 *Department of Periodontology and Oral Implantology, Rural Dental College, Loni, Maharashtra, India*; 2 *Department of Microbiology, Rural Dental College, Loni, Maharashtra, India*

Sir,

Periodontal infection is initiated by specific invasive oral pathogens that colonize dental plaque biofilms on tooth surface, and host immune response to inflammation plays a central role in disease pathogenesis. Periodontal diseases are recognized as infectious processes that require bacterial presence and a host response and are further affected and modified by other local, environmental and genetic factors. Association of periodontal infection with organ systems like cardiovascular system, endocrine system, reproductive system and respiratory system makes periodontal infection a complex multiphase disease.

Periodontitis is defined as an inflammatory disease of supporting tissues of teeth caused by specific microorganisms or groups of specific microorganisms, resulting in progressive destruction of the periodontal ligament and alveolar bone with periodontal pocket formation, gingival recession or both.[1] Periodontal disease is a complex infectious disease resulting from interplay of bacterial infection and host response to bacterial challenge, and the disease is modified by environmental, acquired risk factors and genetic susceptibility. Dental plaque represents a classic example of both a biofilm and a microbial community, in that it displays emer-gent properties, i.e., plaque displays properties that are more than the sum of its constituent members,[2] and microbial communities are ubiquitous in nature and usually exist attached to a surface as a spatially organized biofilm. Recent studies suggest that the environmental heterogeneity generated within biofilms promotes accelerated genotypic and phenotypic diversity that provides a form of “biological insurance” that can safeguard the “microbial community” in the face of adverse conditions, such as those faced by pathogens in the host.[2]

The diversity of bacterial species in the periodontal flora, the variation in composition of floras from individual to individual and the variation in host response to bacterial species are some of the major reasons that the specific etiology of periodontal disease has not been clearly established.[3,4] Bacteria are the primary etiological agent in periodontal disease, and it is estimated that more than 500 different bacterial species are capable of colonizing the adult mouth.[5] Some of the most common organisms associated with periodontal diseases are Porphyromonas gingivalis, Prevotella intermedia, Bacteroides forsythus, Campylobacter rectus and Actinobacillus actinomycetemcomitans, as well as the treponemes.[6] A variety of techniques for analyzing the plaque samples have been developed. These include microscopy, bacterial culture, enzymatic assays, immunoassays, nucleic acid probes and polymerase chain reaction assays,[7] and yet more advanced methods should be explored for more accurate detection of pattern of microbial diversity within the oral cavity.

Recent evidence suggests that periodontal infection may significantly enhance the risk for certain systemic diseases or alter the natural course of systemic conditions; and conditions in which influences of periodontal infection are documented include coronary heart diseases (CHD) and CHD-related events such as angina and infarction, atherosclerosis, stroke, diabetes mellitus; preterm labor, low-birth-weight delivery; and respiratory conditions such as chronic obstructive pulmonary diseases.[1,8] This affiliation does not affect all but definitely affects several. Periodontitis initiates systemic inflammation and can be monitored by inflammatory markers like C-reactive protein or fibrinogen levels.

Periodontitis and periodontal diseases are true infections of the oral cavity. There is an equilibrium that exists between microbial challenge and host's immune response; any alteration to that with addition of other modifying factors is responsible for clinical manifestation of periodontal disease. Pathogens of the subgingival microbiota can interact with host tissues even without direct tissue penetration, and the subgingival microbiota accumulate on the oral cavity to form an adherent layer of plaque with the characteristics of a biofilm. The oral cavity works as a continuous source of infectious agents, and its condition often reflects progression of systemic pathologies. Periodontal infection happens to serve as a bacterial reservoir that may exacerbate systemic diseases.
